# A visual questioning answering approach to enhance robot localization in indoor environments

**DOI:** 10.3389/fnbot.2023.1290584

**Published:** 2023-11-27

**Authors:** Juan Diego Peña-Narvaez, Francisco Martín, José Miguel Guerrero, Rodrigo Pérez-Rodríguez

**Affiliations:** ^1^Intelligent Robotics Lab, Signal Theory, Communications, Telematics Systems, and Computation Department, International Doctoral School, Rey Juan Carlos University, Fuenlabrada, Spain; ^2^Intelligent Robotics Lab, Signal Theory, Communications, Telematics Systems, and Computation Department, Rey Juan Carlos University, Fuenlabrada, Spain

**Keywords:** visual question answering, robot localization, robot navigation, semantic map, robot mapping

## Abstract

Navigating robots with precision in complex environments remains a significant challenge. In this article, we present an innovative approach to enhance robot localization in dynamic and intricate spaces like homes and offices. We leverage Visual Question Answering (VQA) techniques to integrate semantic insights into traditional mapping methods, formulating a novel position hypothesis generation to assist localization methods, while also addressing challenges related to mapping accuracy and localization reliability. Our methodology combines a probabilistic approach with the latest advances in Monte Carlo Localization methods and Visual Language models. The integration of our hypothesis generation mechanism results in more robust robot localization compared to existing approaches. Experimental validation demonstrates the effectiveness of our approach, surpassing state-of-the-art multi-hypothesis algorithms in both position estimation and particle quality. This highlights the potential for accurate self-localization, even in symmetric environments with large corridor spaces. Furthermore, our approach exhibits a high recovery rate from deliberate position alterations, showcasing its robustness. By merging visual sensing, semantic mapping, and advanced localization techniques, we open new horizons for robot navigation. Our work bridges the gap between visual perception, semantic understanding, and traditional mapping, enabling robots to interact with their environment through questions and enrich their map with valuable insights. The code for this project is available on GitHub https://github.com/juandpenan/topology_nav_ros2.

## 1 Introduction

Precision navigation in intricate environments poses a fundamental challenge that engages the interests of researchers and engineers. In contrast to humans, who can adeptly navigate urban landscapes and complex terrains, replicating these abilities in robots is a complex task, especially in dynamic and intricate spaces such as homes and domestic environments. Despite notable advancements in indoor autonomous robot navigation, challenges persist when navigating in environments that lack predefined maps and discernible geometric landmarks. This is particularly evident in settings like office buildings, healthcare facilities, and structures with extensive corridor networks, where classical localization methods can encounter difficulties (Wu et al., 2021).

Robot navigation primarily relies on two critical components: accurate mapping and reliable localization. Mapping involves creating a representation of the environment, typically in the form of either geometric maps (Hornung et al., 2013) or semantic maps (Huang et al., 2023), which help robots understand their surroundings. On the other hand, localization is the process of pinpointing a robot's exact position within this mapped environment. Achieving these components seamlessly presents a substantial challenge. This challenge arises from the fact that many classical methods use particle populations that may converge to incorrect positions, close to the robot's actual position (Wu et al., 2021; Ge et al., 2022). To address this, we propose leveraging the semantic characteristics found in different environments, such as hospital and office buildings, which possess room numbers, door colors, and other features, by harnessing machine learning techniques resulting in a valid robot position hypothesis.

Recent developments in artificial neural network models have showcased their potential for integration into various robotic applications. This is notably evident in the case of Visual Language Models and Large Language Models (Ahn et al., 2022; Huang et al., 2022; Driess et al., 2023; Wu et al., 2023; Xiao et al., 2023), as well as VQA technologies (Deng^*^ et al., 2021; Kamath et al., 2021; Amodeo et al., 2022). These advancements have paved the way for robots to augment classical methods in navigation, localization, mapping, and manipulation by grounding their capabilities in language and visual embedding. By integrating these sophisticated neural network models, robots are endowed with the ability to interact with their environment in a more natural and intuitive manner. They can understand and respond to verbal and visual cues, enabling smoother human-robot interactions. Moreover, these models facilitate a broader scope of robotic tasks. Robots can effectively analyze and interpret visual data from their surroundings, leading to improved decision-making capabilities. They can also leverage language understanding to comprehend instructions and queries from humans, enhancing their adaptability in diverse scenarios. As a result, the integration of artificial neural networks into robotics promises not only increased efficiency in navigation and manipulation but also more user-friendly and effective interactions between robots and humans.

**Figure 1:**
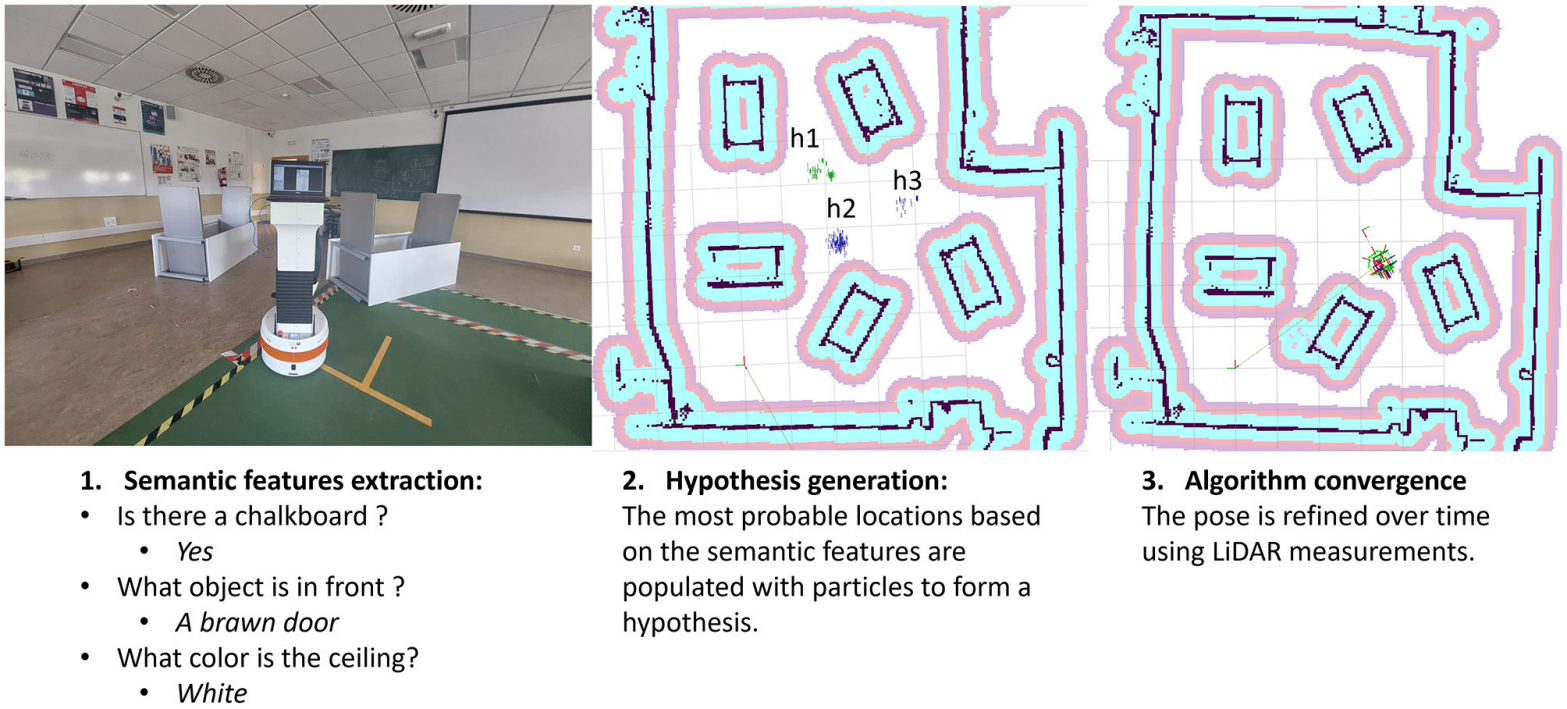
Localization process overview.

This article delves into the capabilities of VQA models (Kamath et al., 2021) in robotics. It shows how these models can add valuable information to traditional costmaps environment representation with semantic insights from statistical environmental models. This work proposes a coarse-to-fine localization paradigm, blending these semantic clues with a classic LiDAR-based method (Garcia et al., 2023) for robust navigation, by changing its map-matching hypothesis generation with our semantic position clues method. Our approach seamlessly fits into the widely used Robot Operating System (ROS 2) framework (Macenski et al., 2022), while extending the capabilities of Navigation 2 (Nav2) (Macenski et al., 2020); an illustration of our method can be seen in Figure 1. This smart integration boosts our approach's reliability, pushing for smarter and more adaptable robots in complex environments. Thus, the main contributions of this work are:

- Introduction of a novel approach for environment representation, leveraging semantic information to bridge the gap between language models and traditional mapping techniques. This representation also enables the incorporation of essential environmental characteristics grounded in natural language.
- Development of an observation model capable of generating robot state hypotheses by inquiring about the surroundings, harnessing the advantages offered by VQA models.
- Comparison and evaluation of the proposed observation model with classical map-matching techniques (Garcia et al., 2023).

In the subsequent sections, we delve into the details of our methodology, present experimental validations, and discuss the implications of our findings for the broader field of robotics.

## 2 Materials and methods

Two pillars in robot navigation are the mapping and localization process. Most of the efforts in this field have focused on particle filtering (Marchetti et al., 2007; Teslić et al., 2010; Pak et al., 2015) and graph optimization methods (Xuexi et al., 2019; Debeunne and Vivet, 2020), relying mostly on LiDAR sensors. One of the most important algorithms is the Adaptive Monte Carlo Localization (AMCL) (Pfaff et al., 2006). It has become a robust approximation for robot navigation applications, and the state-of-the-art robot navigation framework Nav2 (Macenski et al., 2023), implemented in ROS 2 (Macenski et al., 2022), relies on.

Another approach to robot localization is to use multiple hypotheses, improve robot localization precision, and improve localization methods to apply complete uncertainty, such as the kidnapped robot problem (Engelson and McDermott, 1992). Current work on this method has been focused on taking advantage of different sensors with available navigation algorithms, such as Ge et al. (2022), which combines visual features of the environment, identifying spaces in the map tagged with numbers. Another work that employs visual features for hypothesis generation is the one proposed by Yun and Miura (2007), where the lack of GPS precision was compensated with visual information. Furthermore, the incorporation of different types of sensors has been used to produce multiple hypotheses of robot position, such as radio sensors (Xiong et al., 2022), GPS (Schuessler and Axhausen, 2009), visual SLAM (Chan et al., 2018), and Bluetooth Low Energy (BLE) beacons (Zhuang et al., 2016). Map matching techniques have also been successfully implemented for robot localization, which is the case for Garcia et al. (2023), where the map resolution is changed to efficiently scan the whole environment, searching for the hypothesis.

As discussed above, recent advances in natural language processing using neural networks and the robustness of more classical mapping and localization methods have recently generated significant enthusiasm to improve robot navigation (Salas-Moreno et al., 2013; Huang et al., 2023). So far, most efforts have been focused on object detection, adding landmarks to the map based on the object classes. More recently, work has been focused on taking advantage of large pre-trained visual language models, generating novel map representations using the embedded spaces of such models. On the contrary, our methods rely on VQA, enabling a conversational way to improve current map representations.

VQA models have emerged as a means for robots to gather information about their environment by asking questions about what they perceive (Ahn et al., 2022). These models leverage visual inputs such as images and natural language questions to provide answers. Various architectures, including Convolutional Neural Networks (CNNs) and Recurrent Neural Networks (RNNs), have been employed for VQA tasks (Kamath et al., 2021). Our work aligns with this trend, utilizing a VQA model to obtain semantic clues from the environment. By generating questions and interpreting answers, our method enriches the robot's map with valuable insights beyond geometric data.

## 3 Methodology

The proposed localization method employs the discrete Bayes filter algorithm (Cassandra et al., 1996). Central to this approach is the measurement model, which derives its efficacy from semantic features extracted from the environment using a VQA model (Kamath et al., 2021). The robot's belief state, denoted as *bel*(*x*_*t*_), encapsulates its position $x_{t}={\left(x,y,\psi \right)}^{T}$ at time *t*. Here, *x* and *y* represent the coordinates aligned with the map's origin, while ψ signifies a discretized version of the yaw angle.

The computation of this position is facilitated by employing the VQA model. By posing a series of predefined questions in tandem with the most recent camera data, a series of semantic clues, *S* = {*s*_0_, *s*_1_, …, *s*_*T*_}, is generated. Each element *s*_*i*_ within this series corresponds to a vectorized response to queries such as *What is the object that is in front of me?* or *Is there any door?*. By comparing the model's answers with a predefined semantic map *M*, a discretized probability grid emerges, offering a nuanced characterization of the robot's current belief state *bel*(*x*_*t*_).

Within this grid, the greatest values are extracted to form a population of particles. These particles, representative of the semantic clues, are continuously updated using data from the robot's odometry sensor measurements $A=\left\{a_{0},a_{1},...,a_{T}\right\}$, ensuring the robot's state is consistently updated and accurate. This methodology is further enhanced by integrating traditional techniques that utilize LiDAR sensors. The result is a robust localization method with the ability for autonomous self-localization. This capability is achieved by refining the initial visual semantic clues through established methods such as AMCL (Pfaff et al., 2006) or MH-AMCL (Garcia et al., 2023).

### 3.1 Semantic map generation

To generate an appropriate map, we combine classical costmap definitions (Hornung et al., 2013) with semantic information extracted from the environment. This approach enriches a precise geometric map definition, such as the costmap, with valuable semantic insights. Formally, we define the map as *M*_*H*×*W*×*A*×*S*_, where *H* and *W* represent the dimensions of the top-down costmap (*H, W*∈ℝ). *A*∈ℝ indicates the grid discretization of the robot's orientation angle ψ, and *S* is a vector of *n* tuples containing both the answers *t* and the model's scores *q*:


(1) $$\begin{array}{l} S=\left[\left(t_{1,1},q_{1,2},...,t_{1,k},q_{1,k}\right),...,\left(t_{n,1},q_{n,2},...,t_{n,k},q_{n,k}\right)\right] \end{array}$$

Based on the last definition, creating a new map involves three fundamental assumptions: first, a set of pre-defined questions related to the environment are selected; second, the availability of odometry data; and finally, the existence of a costmap, to append semantic information.

In order to generate the costmap where the semantic map will be built, relying on laser and odometry sensors, the mathematical equation used to build a costmap is:


(2) $$\begin{array}{l} p\left(m|Z_{1:t},X_{1:t}\right)=\underset{i}{\prod }p\left(m_{i}|Z_{1:t},X_{1:t}\right) \end{array}$$

The equation denoted as (2) characterizes a probability density function representing the probability of the costmmap's accuracy (*m*), given all available laser sensor measurements (*Z*_1:*t*_) and the robot's positions (*X*_1:*t*_) up to time *t*. The symbol ∏_*i*_ indicates a product taken over each individual element (*m*_*i*_) within the map *m*. Each term *p*(*m*_*i*_|*Z*_1:*t*_, *X*_1:*t*_), signifies the likelihood of a specific costmap element *m*_*i*_ being correct, taking into account all the collected sensor data and robot positions.

Before appending the semantic information into the costmap, a series of questions have to be defined. Those are formulated based on characteristic objects in the environment. For instance, any unique piece of furniture, a distinctive wall color, or a special combination like, *Is the blue chair next to the door?* could be employed to enhance localization performance. More general questions can also be formulated, such as asking about any object in front of the robot. We have determined that employing one or two general questions in conjunction with more specific ones could yield enhanced results.

**Figure 2:**
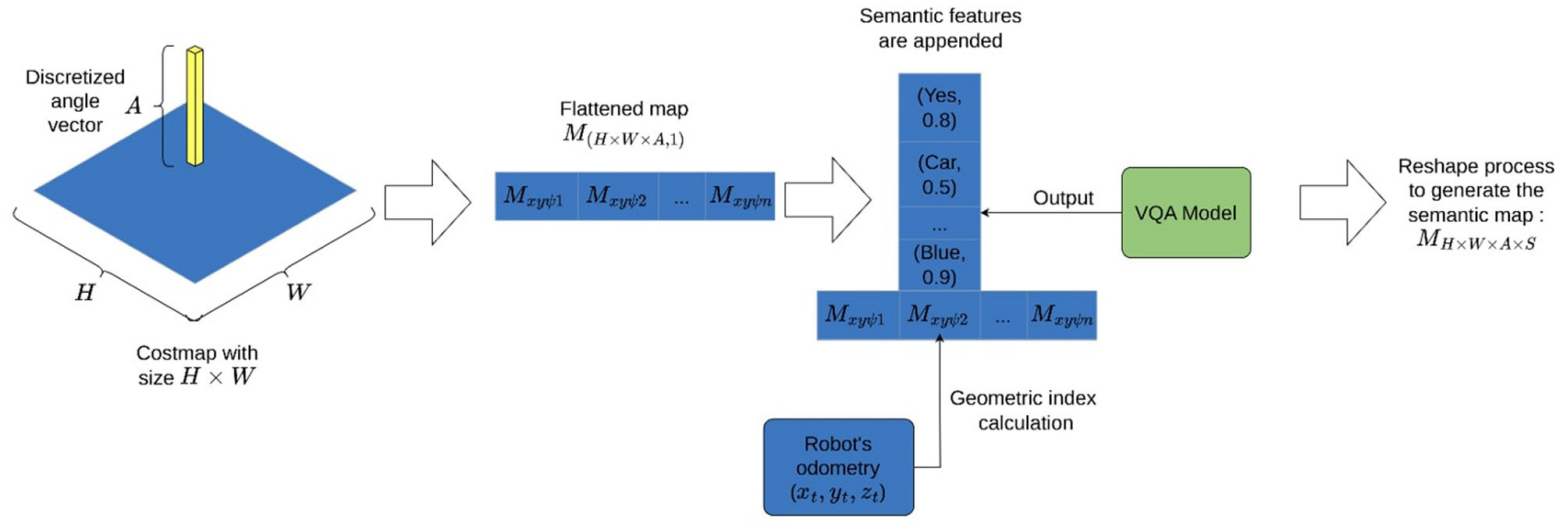
Costmap and angle discretization flattening process to append semantic features.

With the grid map in place, odometry information available, and the questions defined. Extracting semantic information to build the environment involves the robot capturing images while navigating the map. These images are labeled using a geometric index *I*∈ℕ, computed by flattening the first three dimensions of the map *M*_*H*×*W*×*A*_ as shown in Figure 2. For each index, a series of images are captured and stored on disk. It can be seen in Algorithm 1, how the robot uses the odometry information to compute a single index by converting the map data structure into an array of flat indices.

**Algorithm 1:**
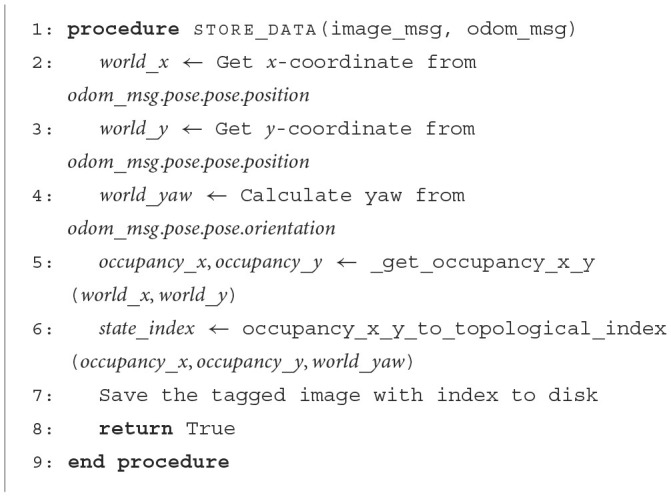
Capture images from environment.

After all images have been collected, the VQA model comes into play. This model is an extension of DETR (Carion et al., 2020), it combines image and text features to enhance object detection. It leverages a shared embedding space, utilizing a cross encoder and a transformer decoder to predict object boxes. The model is pre-trained and evaluated on tasks, including the CLEVR dataset (Johnson et al., 2016).

**Figure 3:**
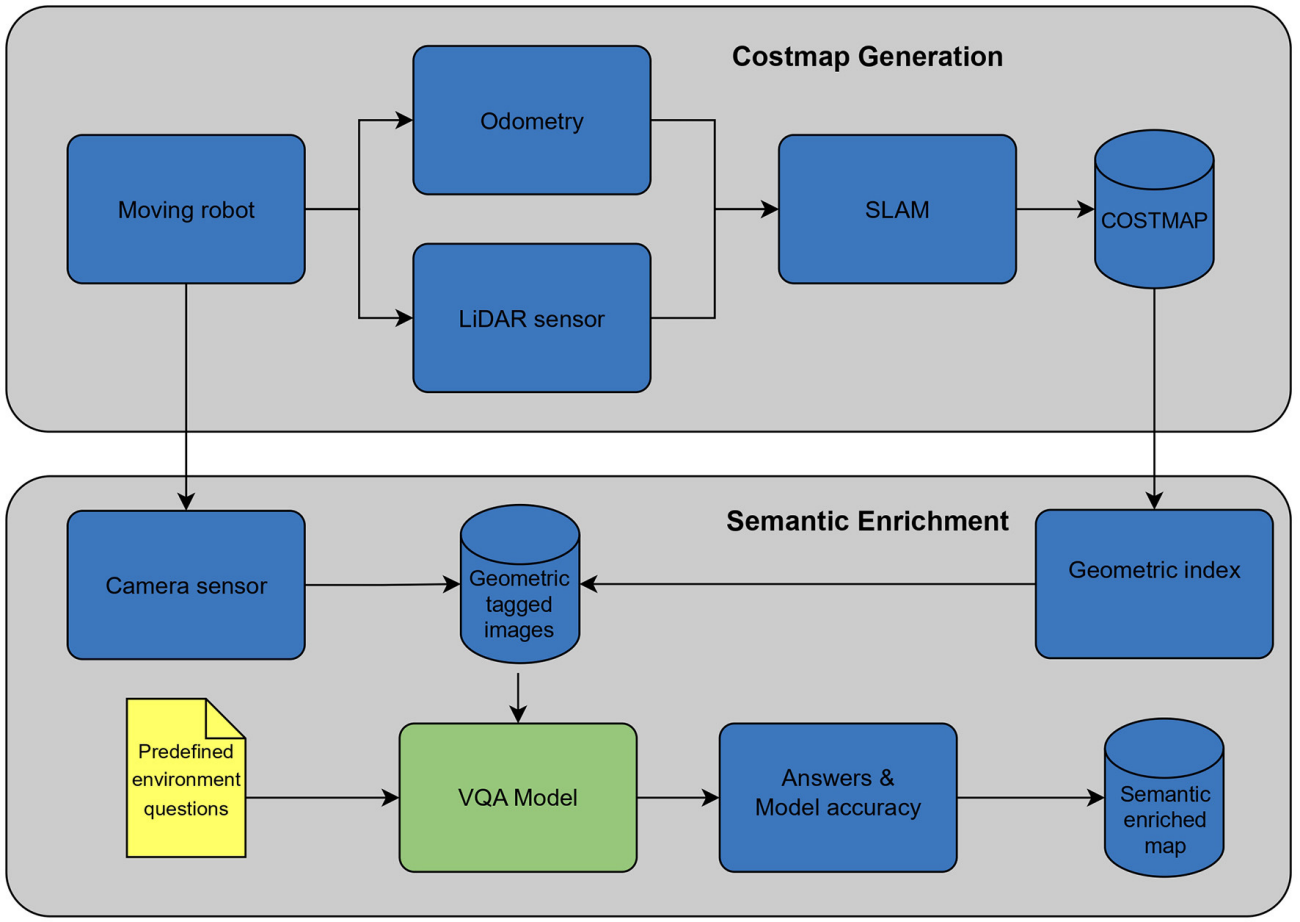
Mapping process overview.

To incorporate semantic features into the map, the VQA model is used for predefined questions and their respective indices. Both the acquired answer and the model score are then added to the costmap. Algorithm 2 provides a clear illustration of how the model is invoked to obtain the answer along with its associated score, which is subsequently appended to the costmap using a unique index to denote its spatial location. In particular, since multiple images are captured for each index, recurring answers are averaged before being integrated into the map. The map generation process is depicted in Figure 3.

**Algorithm 2:**
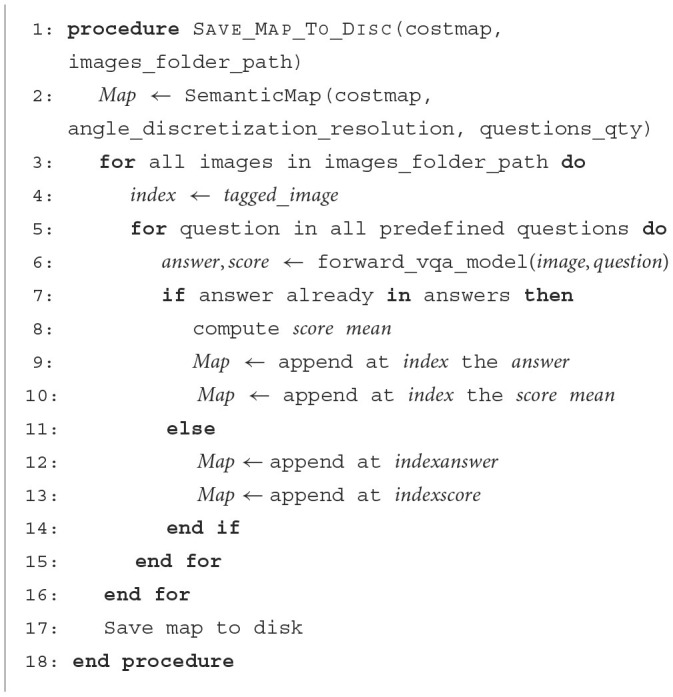
Generate semantic map.

### 3.2 Position hypothesis generation algorithm

The localization algorithm focuses on extracting information from the camera sensor. This process begins with capturing the most recent image taken by the robot, which is then input to a VQA model, prompted with a predefined set of questions *Q*. Those questions explore possible features the robot can use to locate itself, Table 1 shows some question examples that can be used. Once the answers are obtained, a comparison is made against the pre-existing semantic map. Specifically, current answers are looked up in the semantic map, for those that there is a match, we compute the inverse of the distance between the model's current score to the map-recorded values. Additionally, our observation model rewards the possible position if multiple answers align with the map. This is achieved by applying the Bayesian rule and multiplying the probabilities of each answer. The entire process of obtaining a weighted accuracy based on semantic information can be seen in Algorithm 3. It is important to consider that the assumption of independence between answers holds. The top values are clues generated by our measurement model, those are then updated based on the input from actuators. This involves convolving the current distribution on the map to ensure that it remains consistently up-to-date. This forms the initial stage of the localization process. At this stage, the map is populated with potential locations where the robot could be located.

**Table 1:** Sample questions.

**Question examples**
What object is in front?
Is there any human?
What is the ceiling color?
Is there any X object?

**Algorithm 3:**
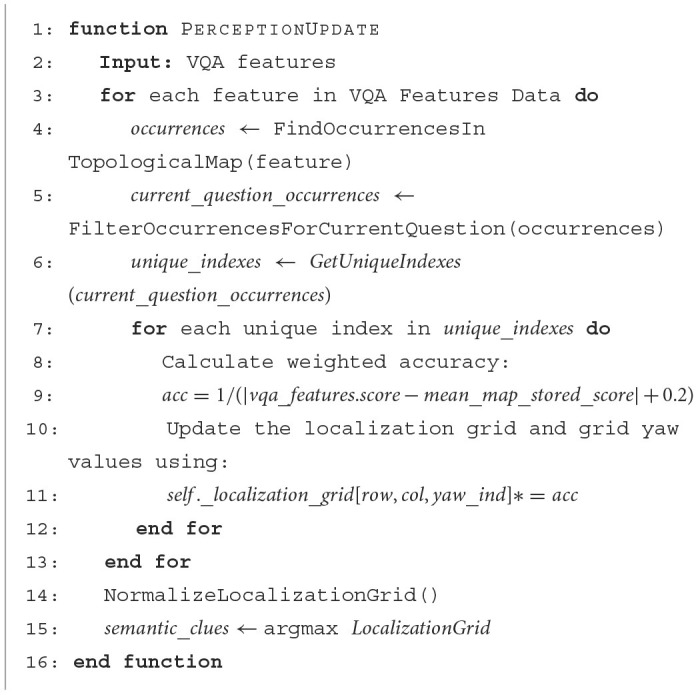
Observation model algorithm.

Subsequently, our approach takes advantage of more traditional methods (Garcia et al., 2023), capitalizing on the precision offered by LiDAR sensors. We establish a particle population for the top four most probable locations, for each population, a predetermined number of particles is generated and continuously updated until convergence. Periodically, the algorithm checks for new particle populations based on the input of the camera sensor. If the existing particles have not yet converged or lack quality, the algorithm generates new particles to replace them.

**Figure 4:**
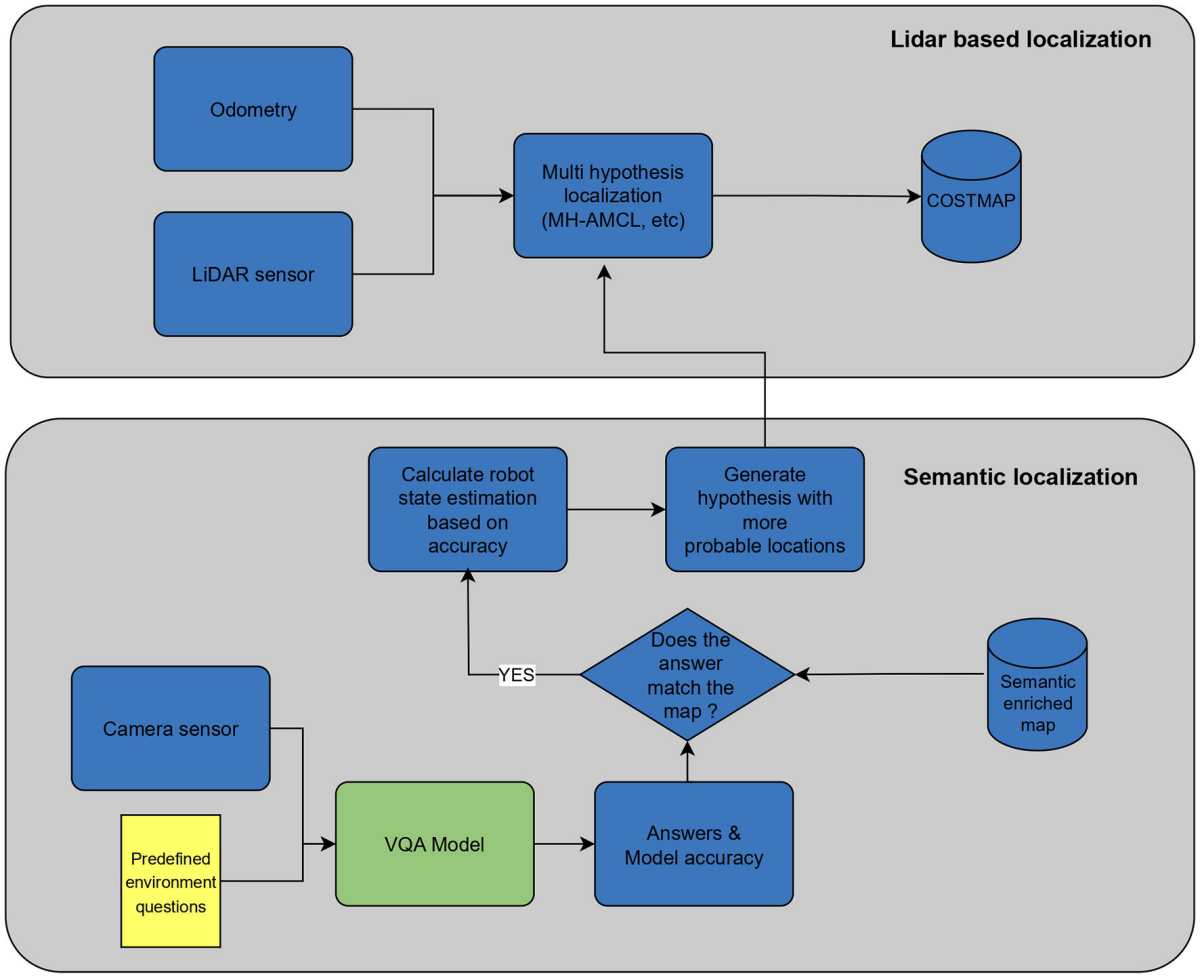
Localization procedure.

This approach supersedes map-matching techniques that often prove computationally intensive for large maps. Unlike the need to iterate through the entire map to generate new particle populations, our approach already incorporates this step by leveraging the VQA model. The overall localization process can be seen in Figure 4.

## 4 Results

To validate our implementation, we performed two experiments in a realistic scenario; the Tiago mobile manipulator robot was used. Since the proposed implementation is based on the reliable and already tested multi-hypothesis localization method (Garcia et al., 2023). We selected Nav2 as the baseline for all the experiments. This framework stands as a pivotal initiative in mobile robotics, offering an advanced system to guide autonomous mobile robots (Macenski et al., 2023). We ensure that Nav2 outputs a reliable robot position using a predefined path. Furthermore, we compared our method with its predecessor, MH-AMCL (Garcia et al., 2023).

The questions used to semantically describe the environment in the experiments are the same in both experiments 4.1 and 4.2. Questions are shown in Table 2.

**Table 2:** Questions used in all experiments.

**Questions**
What object is in front?
Is there a whiteboard?
Is there a fire extinguisher?
Is there a door?
Is there a brown wall?
Is there a chair?
Is there a trash can?

**Figure 5:**
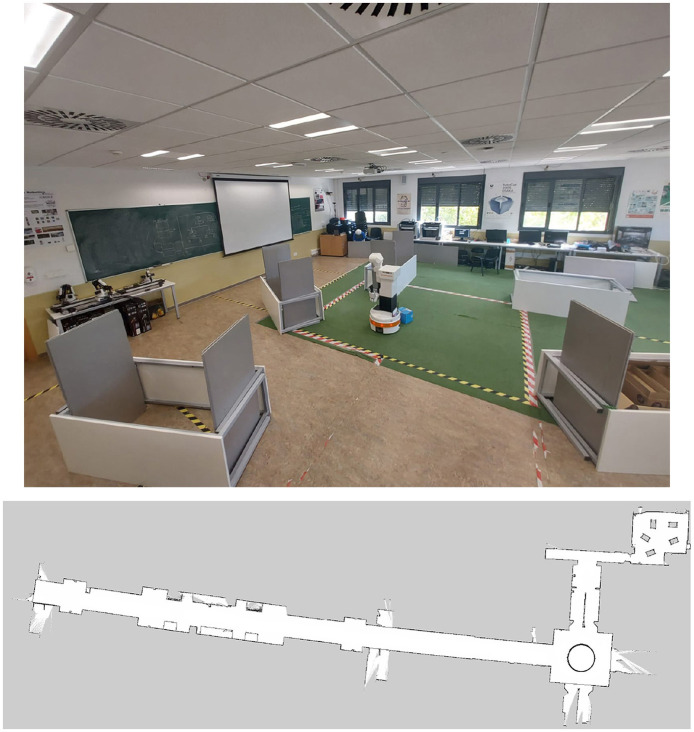
Experiments environment.

All experimental trials were conducted on an entire floor of the building at Rey Juan Carlos University, providing approximately 1200 *m*^2^ for navigation. The environment consists primarily of corridors and a laboratory area, as shown in Figure 5. This setup allowed us to confirm the suitability of our algorithm for expansive spaces. For all experiments, data was captured with the rosbag tool ^1^ using a computer featuring an AMD Ryzen^*TM*^9 7845HX processor, 32GB of RAM, and an NVIDIA RTX 4060 GPU. The proposed experiments are described below.

### 4.1 Experiment 1

This experiment aims to analyze the performance of our method by comparing it with its map-matching precursor method (Garcia et al., 2023) while also evaluating its localization precision by measuring the position error. Three trials were conducted in which the robot had to navigate through a predefined path. In each trial, the robot starts from a known position, and the same path is used throughout all trials, regardless of the method.

First, the baseline position is obtained by following the predefined path using Nav2. Then, using the same path, the localization method is switched to MH-AMCL and, finally, the proposed approach is tested. The same navigation route was repeated three times. For this experiment, the following variables have been measured:

- **Position error**: Absolute error in cm using the Nav2 position as our real value; this error corresponds to the absolute difference between each position component, the *x* and *y* axis, as well as the yaw angle.
- **Particle number and quality**: The quality measurement was introduced by Garcia et al. (2023) and was used to determine the best particle population by averaging the likelihood of the particles with respect to the last sensory perception. This quality metric assesses the correspondence between predicted and current laser scans by computing the rate of matched laser beams. Introducing this metric enables a quantitative evaluation of particle alignment with laser data. Specifically, the quality can be calculated as follows:

(3) $$\text{Quality}(P_{t})=\frac{\sum_{j=0}^{n}p_{j}^{t}.h}{\left|Z_{t}|\cdot |P_{t}\right|}$$

- $p_{j}^{t}.h$ represents the matching laser hits associated with each particle $p_{j}^{t}$ in the population *Pt*.
- *n* represents the total number of particles in the population.
- |*Zt*| represents the total number of sensor readings in the observation *Zt*.
- |*Pt*| represents the total number of particles in the population.

**Figure 6:**
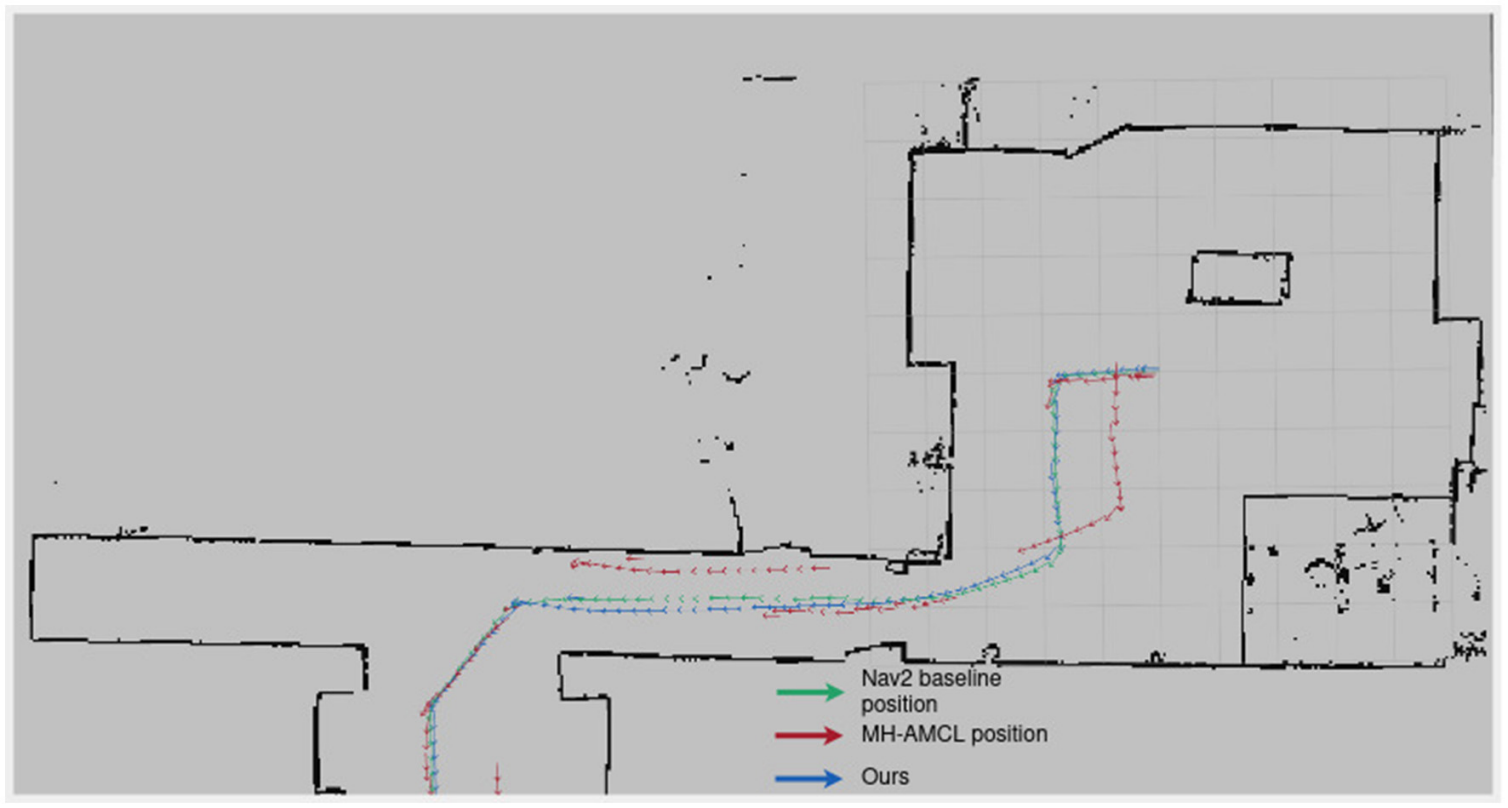
Followed path by each method in Experiment 1.

In Figure 6, the paths taken by the compared methods are seen. The MH-AMCL method deviates in several parts of the route. In contrast, our method consistently follows the intended positions.

**Figure 7:**
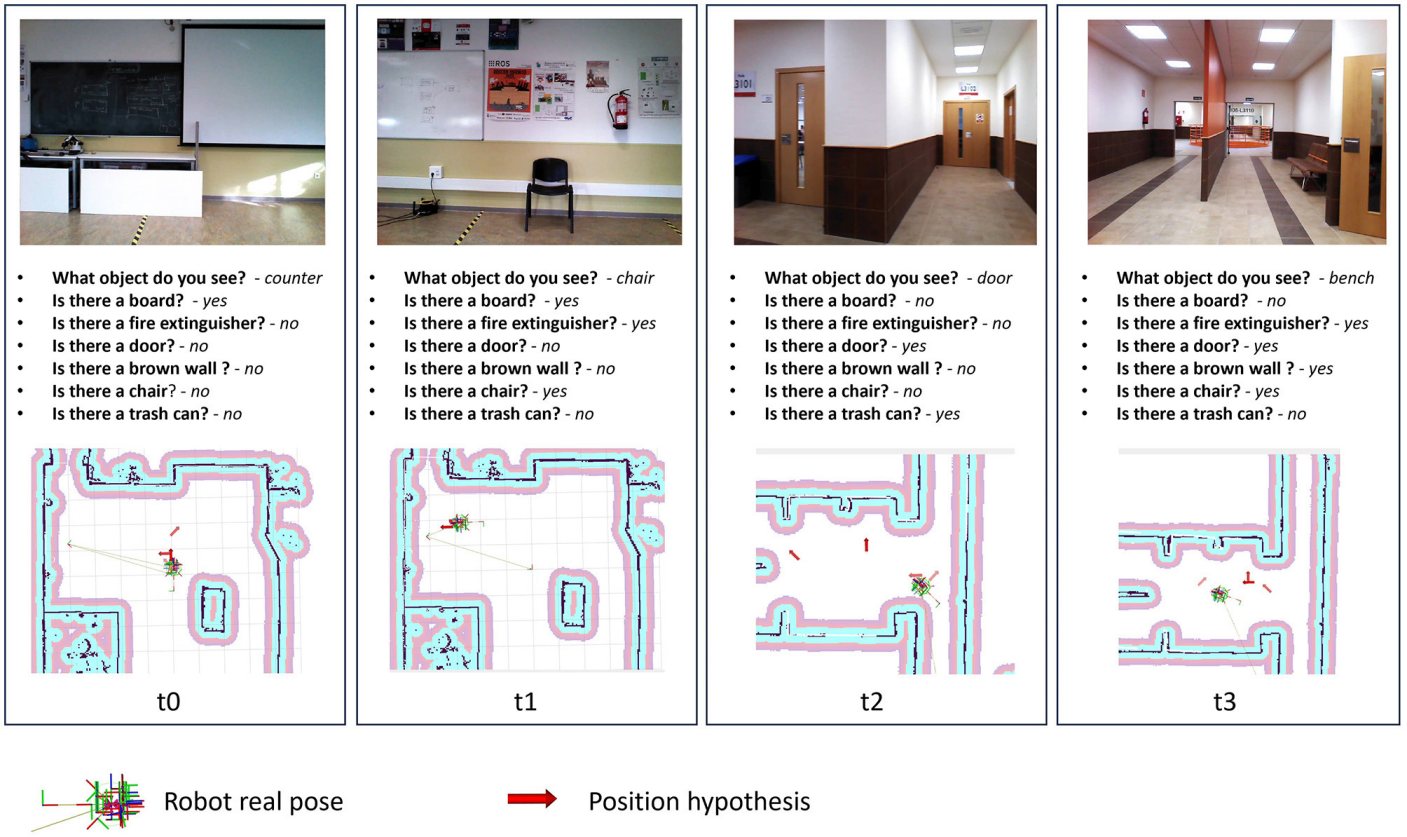
Proposed method introspection for the experiment 1. Position hypothesis are the top values generated by Position hypothesis generation process.

One of the key factors contributing to our algorithm's ability to converge to an accurate position is its adeptness in generating precise position hypotheses. This proficiency is notably attributed to the meticulous formulation of environmental queries during the mapping process. As depicted in Figure 7, our hypothesis generation method consistently yields positions in close proximity to the actual robot position. The image vividly demonstrates how the input images received by the robot, in conjunction with the output of the VQA model, contribute to the generation of accurate robot position hypotheses.

**Figure 8:**
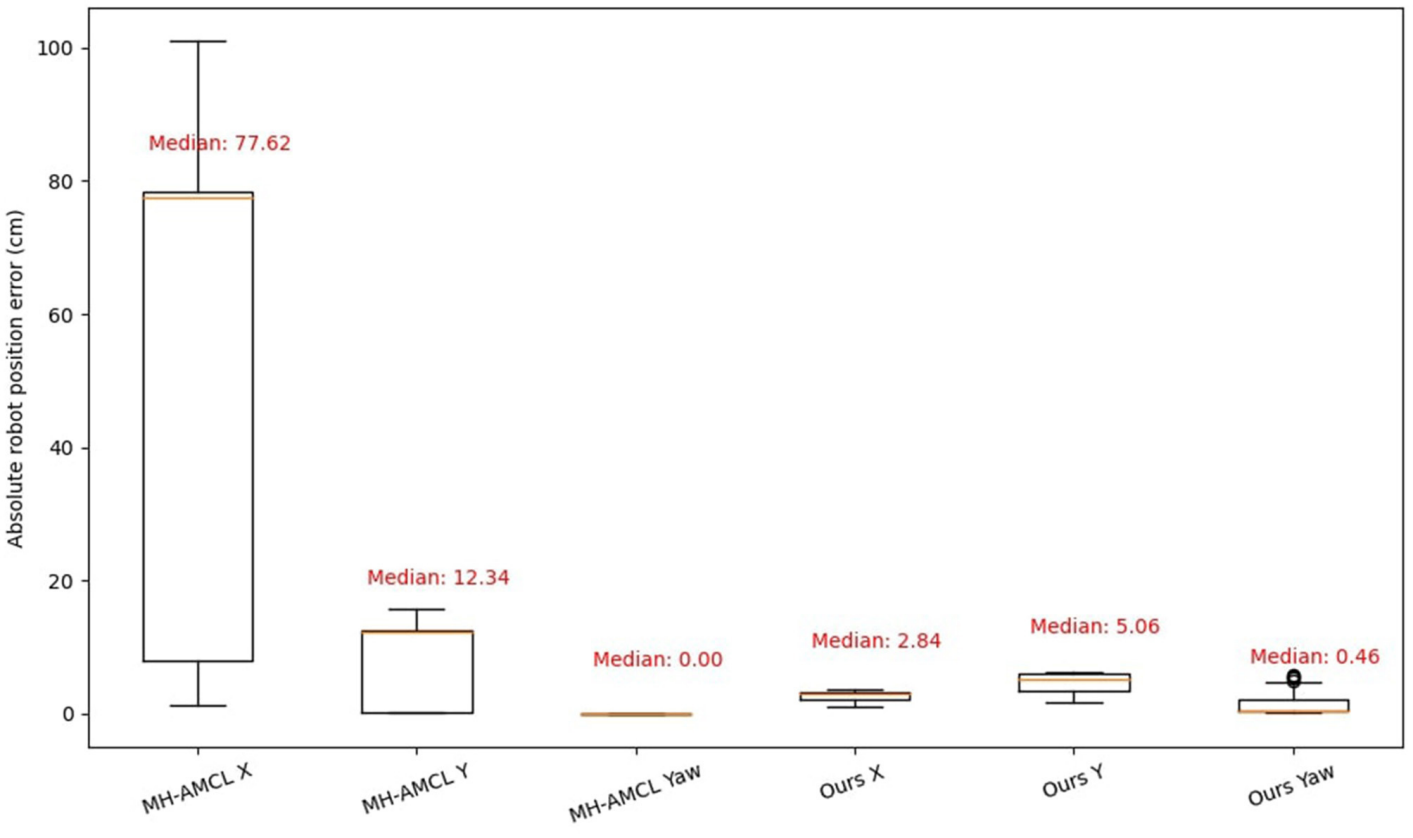
Absolute error position in X, Y axis and Yaw direction.

Figure 8 shows the position error (in cm) separated by the three main directions, x, y, and yaw. Both MH-AMCL and our algorithm are presented in the figure. For the proposed experiments, our method achieved better position estimation in both X and Y directions. However, the MH-AMCL algorithm achieves better results on the yaw angle. The observed error can be attributed to the fact that, although the MH-AMCL method may diverge in certain segments of the path, as illustrated in Figure 6, it consistently maintains the correct orientation. Overall, our method stands out in precision for large environments.

**Figure 9:**
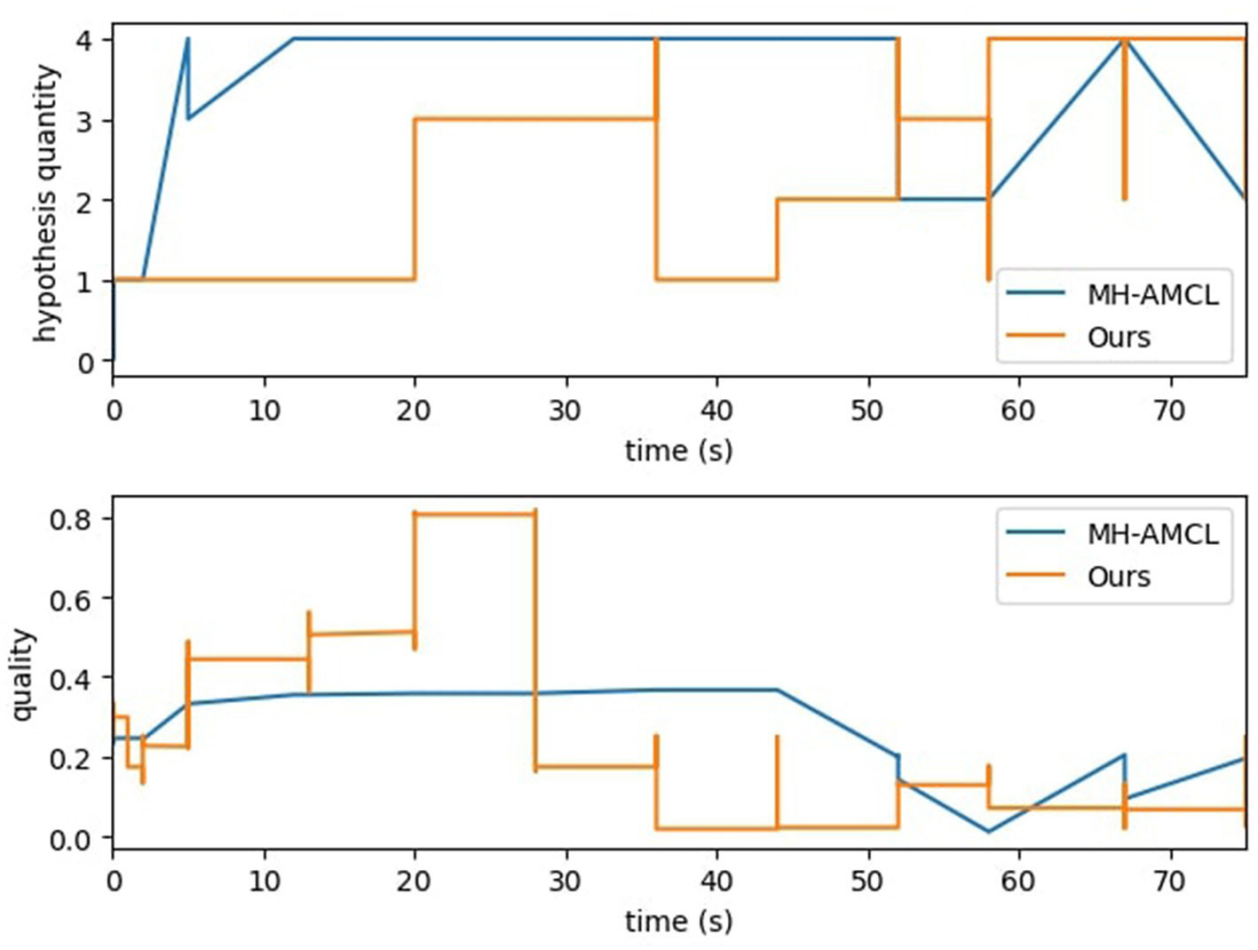
Particle analysis for MH-AMCL and proposed method.

Figure 9 displays the trends of the MH-AMCL particles compared to our algorithm. The MH-AMCL approach shows a direct relation between the quality of the particles and the hypothesis generated. On the other hand, our method achieves good-quality particles in the first half of the experiment, although the number of hypotheses is smaller than the MH-AMCL algorithm, as seen in Figure 9. In both methods, after 50 s of experiment execution, the number of particles starts to oscillate between 2 and 4. The quality trend of our proposed method exhibits a discrete pattern. Unlike the compared method, MH-AMCL, scans the entire map to identify geometric similarities from the laser scan, leading to similar geometric position hypotheses and, consequently, similar quality outputs. Conversely, our approach generates hypotheses based on semantic features, which are more likely to vary in geometry, resulting in a divergence in the quality metric.

### 4.2 Experiment 2

This experiment aims to gauge the algorithm's robustness for self-localization. To assess this, the robot's position is intentionally altered every 30 s, and we measured the recovery time as well as the success rate. The experiment was repeated three times and values presented in Table 3 represent the average. The Nav2 baseline was excluded from this experiment as it does not address the kidnapped robot problem, and its robustness is insufficient for a meaningful comparison in this specific scenario.

**Table 3:** Recovery time.

	**MH-AMCL**	**Ours**
Recovery time (s)	0.028001	0.016917
Success rate	100%	100%

**Figure 10:**
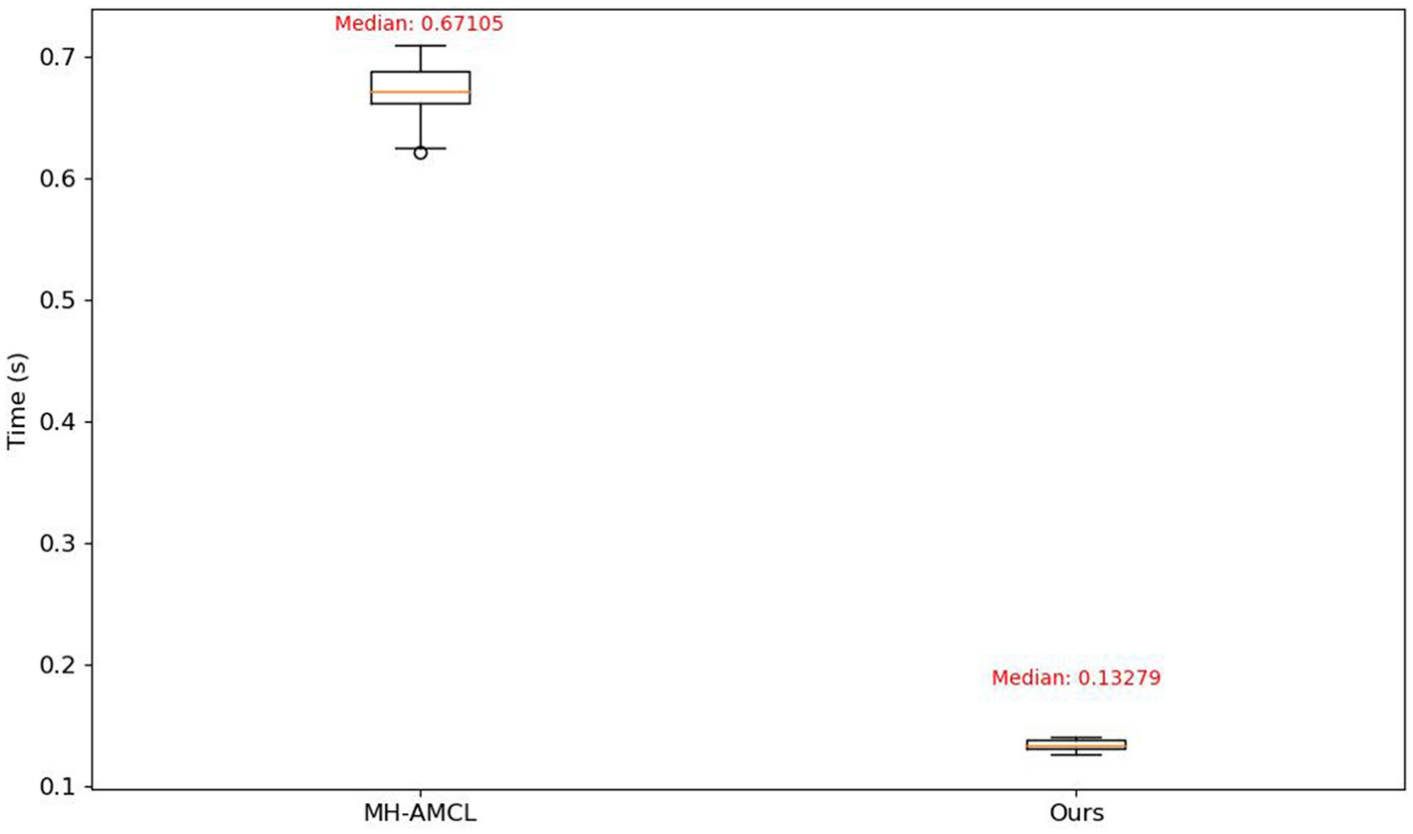
Time comparison of localization algorithm execution.

In Table 3, it is shown how the proposed method improves the recovery time while maintaining the success rate, this While MH-AMCL generates hypotheses by scanning the entire cost map, our method produces hypotheses based solely on the current matched output model questions with the semantic map. This approach allows for more efficient and targeted hypothesis generation, enhancing the overall localization process. This difference can be also seen at Figure 10 where the computing time of each method is shown.

## 5 Discussion

In this paper, we present a novel methodology to improve robot localization in indoor settings using position hypotheses derived from the potency of Visual Question Answering VQA techniques. This approach addresses the challenges associated with achieving accurate mapping and offers reliable localization when integrated with traditional methods, especially in complex and large environments. In particular, these challenges become pronounced in spaces characterized by symmetry, which are frequently encountered in residential, office, and healthcare-related buildings. We have enriched conventional mapping techniques by seamlessly integrating semantic insights derived from a VQA model. This integration has yielded a resilient and versatile approach to position hypothesis generation for robot localization.

Our experiments demonstrated the effectiveness of our approach in improving robot localization precision. In Experiment 1, our method showcased superior performance in position estimation and particle quality compared to the MH-AMCL algorithm. This indicates the potential of our VQA-based approach to achieve accurate and efficient self-localization in large environments.

Furthermore, Experiment 2 highlighted the robustness of our algorithm in recovering from intentional position alterations. The low recovery time and 100% success rate underscore the reliability of our method even in scenarios with deliberate disruptions.

Our work contributes to robot navigation by bridging the gap between visual sensing, semantic understanding, and traditional mapping techniques. By incorporating VQA models, we offer robots the capability to interact with their environment through questions, enriching their map with valuable insights beyond geometric data.

## Data availability statement

The original contributions presented in the study are included in the article/Supplementary material, further inquiries can be directed to the corresponding author.

## Author contributions

JP-N: Software, Validation, Writing—original draft, Methodology, Visualization, Investigation, Writing—review & editing. FM: Conceptualization, Resources, Software, Supervision, Validation, Writing—review & editing. JG: Visualization, Writing—review & editing. RP-R: Validation, Visualization, Writing—review & editing.

## Footnotes

^1^ https://github.com/ros2/rosbag2

## Glossary

### Visual question answering

A field of artificial intelligence that involves training models to understand and respond to questions about visual data, typically images or videos. It combines computer vision techniques (to analyze images) with natural language processing (to understand and answer questions). In the context of the paper, VQA is used to gather information about the robot's environment by asking questions about what it perceives.

### Semantic features/insights

Meaningful information and understanding derived from the analysis of data in a way that considers the context and meaning of the elements involved. In the context of the paper, it involves extracting meaningful information about the environment from VQA models answers, those can go beyond just geometric data, incorporating elements like object and rooms identification, colors, etc.

### Monte carlo localization

A probabilistic technique used in robotics for estimating the position of a robot within an environment. It works by maintaining a large number of hypothetical positions (particles) and updating their likelihood based on sensor measurements. Over time, as the robot moves and receives sensor data, the particles converge to a more accurate estimate of the robot's position.

### Robot mapping

Creating a representation of the environment in which a robot operates. This representation is typically in the form of a map that helps the robot understand and navigate its surroundings. Mapping can be done using various sensors, such as LIDAR, cameras, or other types of detectors, and it can include both geometric information (like distances and angles) as well as semantic information (like object labels or room identifications).
